# Shoulder Lesions Do Not Increase Inflammatory Biomarkers in Patients Undergoing Surgery for Glenohumeral Instability: An Exploratory Study

**DOI:** 10.1155/2022/4220356

**Published:** 2022-02-27

**Authors:** Jonathan S. Yu, David M. Dare, Daniel Edon, Alec L. Sinatro, Dylan C. Sarver, Scott Rodeo, Joshua S. Dines, Christopher L. Mendias

**Affiliations:** ^1^Hospital for Special Surgery, New York, NY, USA; ^2^Weill Cornell Medicine, New York, NY, USA; ^3^Raleigh Orthopedic Clinic, Raleigh, NC, USA; ^4^Albert Einstein College of Medicine, New York, NY, USA; ^5^Johns Hopkins University School of Medicine, Baltimore, MD, USA; ^6^Department of Orthopaedic Surgery, University of Michigan, Ann Arbor, MI, USA

## Abstract

Circulating protein biomarkers have demonstrated utility as a diagnostic tool in predicting musculoskeletal disease severity, but their utility in the evaluation of shoulder lesions associated with shoulder instability is unknown. Thus, the purpose of this exploratory study was to determine whether preoperative biomarkers of cartilage turnover and inflammation are associated with specific shoulder lesions in shoulder instability. Thirty-three patients (29.9 ± 9.4 years of age, 4.5 ± 4.7 dislocations) undergoing surgical treatment for shoulder instability were assessed for the presence or absence of associated shoulder lesions. Biomarkers including cartilage oligomeric matrix protein (COMP), C-reactive protein (HS-CRP), interleukin-8 (IL-8), and macrophage inflammatory protein-1*β* (MIP-1b) were collected at the time of surgery. Patients with Hill-Sachs lesions had a 31% increase in COMP plasma levels (*p*=0.046). No other significant differences were observed for COMP, HS-CRP, IL-8, and MIP-1b with any shoulder lesion including Hill-Sachs lesions, capsular injuries, bony Bankart lesions, and SLAP lesions. In conclusion, inflammatory biomarkers including HS-CRP, IL-8, and MIP-1b were not associated with specific shoulder lesions, while biomarkers of cartilage turnover (COMP) were only elevated in Hill-Sachs lesions. These findings suggest that these biomarkers may have limited utility as prognostic indicators in patients with shoulder instability, though large-scale and longitudinal studies are still necessary.

## 1. Introduction

Glenohumeral instability is a common injury among young and active populations. With an incidence rate of 24 per 100,000 people-years in North America, glenohumeral dislocations are commonly associated with intra-articular lesions such as Bankart lesions, Hill-Sachs lesions, capsular injuries, bony Bankart lesions, and superior labral tear from anterior to posterior (SLAP) lesions [1, 2]. Bankart lesions can occur in over 90% of shoulder instability events, while Hill-Sachs lesions are associated with approximately 40% to 90% of all anterior shoulder instability events, approaching close to 100% with recurrent anterior glenohumeral instability [3–6]. These lesions do not always manifest clinically, and physical examination tests have varying levels of diagnostic accuracy. Regardless, these shoulder lesions can compromise the treatment of shoulder instability if not diagnosed [7].

Circulating biomarkers of cartilage turnover and general inflammation play a role in tracking osteoarthritis progression and have been studied in anterior cruciate ligament (ACL) tears and femoroacetabular impingement (FAI) [8–10]. Previous research into serum cartilage biomarkers associated with shoulder instability revealed no significant differences in preinjury biomarkers in shoulder instability patients compared to controls [11]. However, to date, no study has examined the relationship between circulating biomarkers and associated structural lesions in shoulder instability. Furthermore, the measurement of biomarkers from blood or joint fluid currently does not play a role in the evaluation of shoulder instability.

The purpose of this exploratory study was to determine the potential utility of biomarkers as diagnostic injury predictors or as markers to track injury progression in the setting of shoulder instability. Specifically, we sought to delineate the relationship between biomarkers of cartilage turnover and inflammation and associated lesions in shoulder instability patients by evaluating cartilage oligomeric matrix protein (COMP) as a cartilage turnover marker and C-reactive protein using a high sensitivity assay (HS-CRP), IL-8, and macrophage inflammatory protein 1*β* (MIP-1b) as general inflammatory markers. We hypothesized that shoulder instability patients with associated lesions such as Hill-Sachs lesions or capsular injuries would have elevated biomarker levels of cartilage turnover and inflammation compared to shoulder instability patients without these corresponding lesions.

## 2. Materials and Methods

### 2.1. Subjects

This exploratory study was approved by the local Institutional Review Board. All subjects provided informed consent prior to participating in the study. Subjects were recruited from male patients treated on the sports medicine service. The inclusion criteria included a history of 1 or more traumatic anterior unidirectional shoulder dislocations requiring reduction, 17 to 60 years of age, male, and agreement to undergo surgical stabilization. Subjects were excluded if they had a history of prior shoulder stabilization surgery, major trauma to other joints, chronic joint pain, other major medical illnesses, underlying musculoskeletal conditions, or history of multiple traumas. All patients included in the study underwent surgical repair with preoperative imaging. None of the patients underwent a course of conservative therapy beforehand. The presence or absence of a Bankart lesion, Hill-Sachs lesion, capsular injury, bony Bankart, and/or SLAP lesion along with instability grading at the time of surgery (examination under anesthesia [EUA]) was determined by board-certified and fellowship-trained orthopedic surgeons at the time of surgery. For instability grading during examination under anesthesia, gradings of 3+ were defined as “dislocators” while gradings of 1+ or 2+ were defined as “subluxers.” Time from instability event to blood draw evaluations was not collected consistently across the sample of patients and was thus not included as the part of the study.

### 2.2. Patient-Reported Outcome Measures

Patient-reported number of total dislocations along with patient-reported outcome measurements including ASES shoulder score and Penn shoulder score were collected preoperatively from all patients. The Penn shoulder score [12] is a 100-point scale consisting of 3 subscales: pain, satisfaction, and function that include several scales initially reported in the American Shoulder and Elbow Surgeons (ASES) Shoulder Score survey [13]. A total Penn shoulder score of 100 indicates the optimal function, pain, and satisfaction with the shoulder, while a 0 indicates the worst possible patient-reported outcome.

### 2.3. Measurement of Biomarkers

At the time of surgery, approximately 4 mL of blood was collected from an antecubital vein into a K_2_-EDTA tube. Blood was spun down at 1000*g* for 10 minutes, and plasma was collected and stored at –80°C until use. Protein biomarkers were measured from plasma as previously described [14, 15]. Cartilage oligomeric matrix protein (COMP), R&D Systems, Minneapolis (MN), and high sensitivity C-reactive protein (HS-CRP), Calbiotech, Spring Valley, CA were measured using ELISAs. Granulocyte colony stimulating factor (G-CSF), granulocyte macrophage colony stimulating factor (GM-CSF), interferon *γ* (IFN*γ*), interleukin 1*β* (IL-1*β*), IL-2, IL-4, IL-5, IL-6, IL-7, IL-8, IL-10, IL-12, IL-13, IL-17, monocyte chemoattractant protein 1 (MCP1), macrophage inflammatory protein 1*β* (MIP-1b), and tumor necrosis factor *α* (TNF*α*) were measured using a multiplex Luminex assay (bioplex 17 cytokine, Bio-Rad, Hercules, CA). With the exception of COMP, HS-CRP, IL-8, and MIP-1b, most subjects had levels of cytokines below the detection limit of the assay. Thus, only plasma COMP, HS-CRP, IL-8, and MIP-1b were included in the analysis. Synovial fluid was collected at the time of surgery as well. However, the volume of collectable synovial fluid was too low to conduct biomarker assays.

### 2.4. Statistical Analyses

Biomarker results are presented as mean ± 95% CI. Demographic results are presented as mean ± SD. Prism 8.0 (GraphPad Software, La Jolla, CA) was used to analyze results. Differences between groups were tested using student's *t* tests and chi-squared tests, and *p* values ≤0.05 were considered significant. Cohen's *d* was calculated as a measure of effect size, and the following guidelines were used in the interpretation of effect size: very small 0.01, small 0.20, medium 0.50, and large 0.80 [16]. Pearson's correlation coefficients (*R*) and *p* values were calculated for linear regression analyses.

## 3. Results

A total of *N* = 33 patients were enrolled in this exploratory study and provided blood for COMP and CRP biomarker testing. Of these subjects, 24 patients provided additional samples for IL-8 and MIP-1b biomarker testing. The age of subjects was 29.9 ± 9.4 years (range 17.0 to 50.1 years), and the BMI of subjects was 25.4 ± 3.3 kg/m^2^ (range 20.7 to 37.1 kg/m^2^). The average number of dislocations was 4.5 ± 4.7 (range 1 to 20). Shoulder lesions in the affected shoulder were evaluated and confirmed under arthroscopic examination, with Bankart lesions as the highest prevalence lesion (Table 1). Co-occurrence of shoulder lesions in the patient population was high, andthe majority of patients with Hill-Sachs lesions, capsular injury, bony Bankart lesion, or SLAP lesion also had a concurrent Bankart lesion.

**Figure 1:**
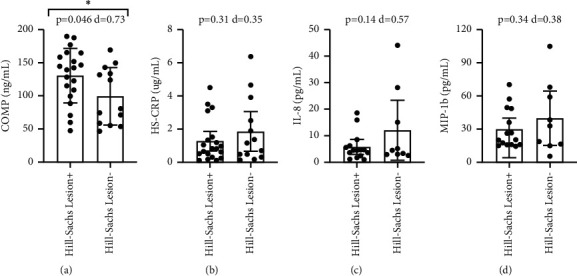
Title: Circulating Biomarker Levels by Hill-Sachs Lesion. Legend: Circulating biomarker levels in patients with and without Hill-Sachs lesions by COMP (a), HS-CRP (b), IL-8 (c), and MIP-1b (d). Differences were tested with a *t*-test and Cohen's d is reported as a measure of effect size. ^*∗*^ denotes a *p*-value <0.05.

**Figure 2:**
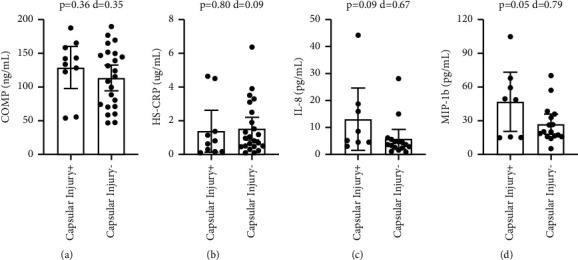
Title: Circulating biomarker levels by capsular injury. Legend: Circulating biomarker levels in patients with and without capsular injury by COMP (a), HS-CRP (b), IL-8 (c), and MIP-1b (d). Differences were tested with a *t*-test and Cohen's d is reported as a measure of effect size. ^*∗*^ denotes a *p*-value <0.05.

**Figure 3:**
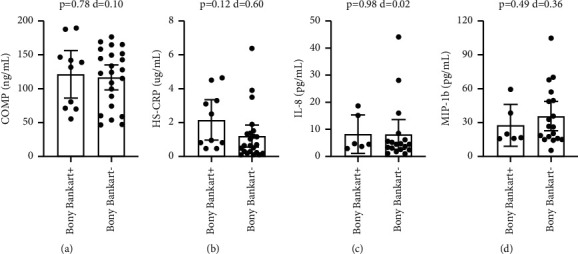
Title: Circulating biomarker levels by bony bankart lesion. Legend: Circulating biomarker levels in patients with and without bony Bankart lesions by COMP (a), HS-CRP (b), IL-8 (c), and MIP-1b (d). Differences were tested with a *t*-test and Cohen's d is reported as a measure of effect size. ^*∗*^ denotes a *p*-value <0.05.

**Figure 4:**
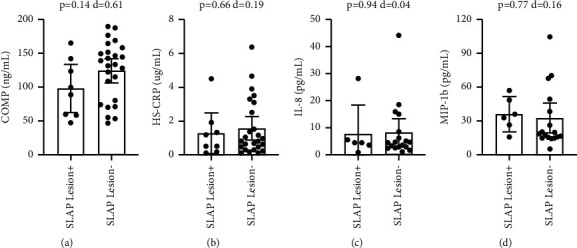
Title: Circulating biomarker levels by SLAP lesion. Legend: Circulating biomarker levels in patients with and without SLAP lesions by COMP (a), HS-CRP (b), IL-8 (c), and MIP-1b (d). Differences were tested with a *t*-test and Cohen's d is reported as a measure of effect size. ^*∗*^ denotes a *p*-value <0.05.

**Figure 5:**
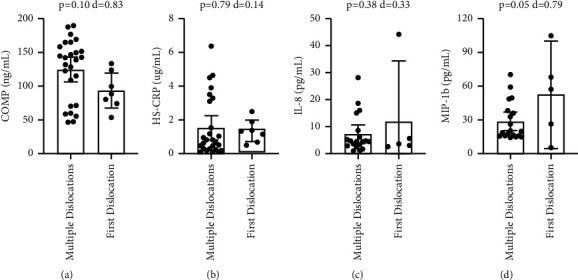
Title: Circulating biomarker levels by multiple dislocations versus first dislocation. Legend: Circulating biomarker levels in patients with multiple dislocations or first-time dislocation by COMP (a), HS-CRP (b), IL-8 (c), and MIP-1b (d). Differences were tested with a *t*-test and Cohen's d is reported as a measure of effect size. ^*∗*^ denotes a *p*-value <0.05.

With Bankart lesions occurring in 91% of the study population, the non-Bankart lesion sample was not large enough for biomarker comparison. Patients with Hill-Sachs lesions had significantly higher circulating levels of COMP (p=-0.046), with a medium-large effect size (*d* = 0.73) (Figure 1(a)). Comparing patients with and without Hill-Sachs lesions, there were no significant differences in CRP (*p*=0.31), IL-8 (*p*=0.14), or MIP-1b (*p*=0.34) (Figures 1(b)–1(d)). Comparing patients with and without capsular injuries involving anterior labral periosteal sleeve avulsions, there were no significant differences in COMP (*p*=0.36), CRP (*p*=0.80), IL-8 (*p*=0.09), or MIP-1b (*p*=0.05) (Figure 2). Comparing patients with and without bony Bankart lesions, there were no significant differences in COMP (*p*=0.78), CRP (*p*=0.12), IL-8 (*p*=0.98), or MIP-1b (*p*=0.49) (Figure 3). Comparing patients with and without SLAP lesions, there were no significant differences in COMP (*p*=0.14), CRP (*p*=0.66), IL-8 (*p*=0.94), or MIP-1b (*p*=0.77) (Figure 4). Comparing patients with multiple shoulder dislocations versus patients with a first-time dislocation, there were no significant differences in COMP (*p*=0.10), CRP (*p*=0.79), IL-8 (*p*=0.38), or MIP-1b (*p*=0.05) (Figure 5).

**Figure 6:**
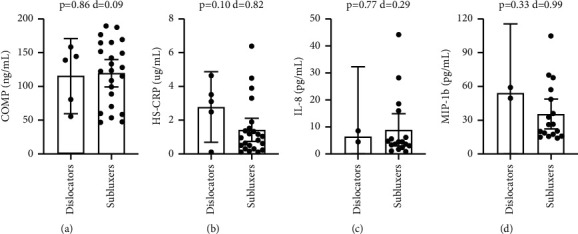
Title: Circulating biomarker levels by examination under anesthesia instability grading. Legend: Circulating biomarker levels in patients with 3+ EUA grading (dislocators) vs. 1+ or 2+ EUA grading (subluxers) COMP (a), HS-CRP (b), IL-8 (c), and MIP-1b (d). Differences were tested with a *t*-test and Cohen's d is reported as a measure of effect size. ^*∗*^ denotes a *p*-value <0.05.

**Figure 7:**
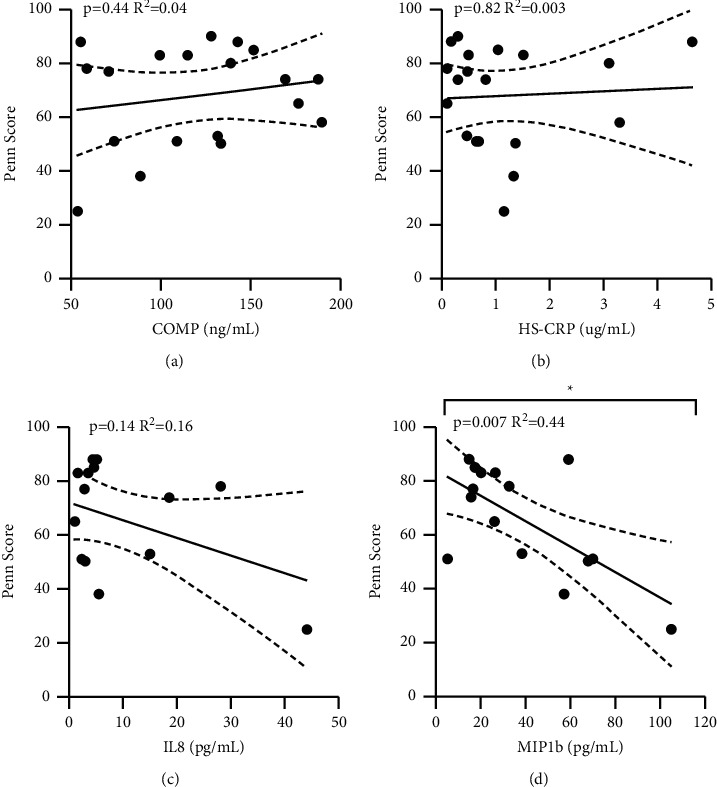
Title: Circulating biomarker levels by ASES shoulder score. Legend: Circulating biomarker levels in patients associated with preoperative ASES Shoulder Scores by COMP (a), HS-CRP (b), IL-8 (c), and MIP-1b (d). Associations were tested with a linear regression and *R*^2^ is reported. ^*∗*^ denotes a *p*-value <0.05.

**Figure 8:**
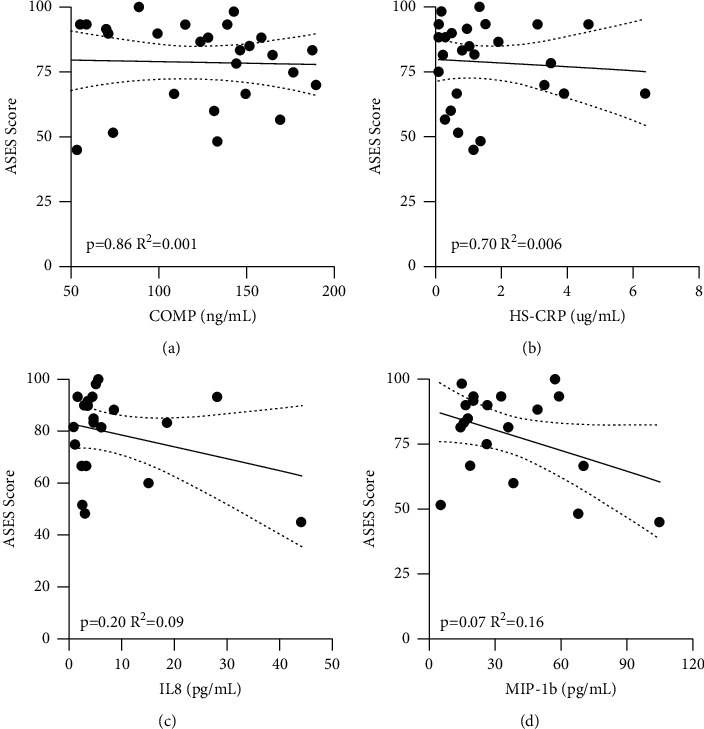
Circulating Biomarker Levels by ASES Shoulder Score. Circulating biomarker levels in patients associated with preoperative ASES Shoulder Scores by COMP (a), HS-CRP (b), IL-8 (c), and MIP-1b (d) Associations were tested with a linear regression and R 2 is reported. ^*∗*^denotes a *p*-value <0.05.

No significant correlations were found between COMP (*p*=0.86), CRP (*p*=0.10), IL-8 (*p*=0.77), or MIP-1b (*p*=0.33) and instability grading at the time of surgery (exam under anesthesia) (Figure 6). Patients with an increase in levels of MIP-1b had lower Penn shoulder scores (*p*=0.007, *R*^2^ = 0.44) (Figure 7(d)). The Penn shoulder score minimally clinically important difference (MCID) for the improvement of 11.4 points [12] was associated with a 23.1 pg/ml decrease in MIP-1b biomarker levels (average MIP-1b levels: 33.4 ± 23.6 pg/ml, range 5.2 to 104.8 pg/ml). No significant correlations were found between COMP (*p*=0.44), CRP (*p*=0.82), or IL-8 (*p*=0.14) and Penn shoulder scores (Figures 7(a)–7(c)). No significant correlations were found between COMP (*p*=0.86), CRP (*p*=0.70), IL-8 (*p*=0.20), or MIP-1b (*p*=0.07) and ASES shoulder scores (Figure 8).

## 4. Discussion

The primary objective of this exploratory study was to determine the potential utility of biomarkers as diagnostic injury predictors or as markers to track injury progression in the setting of shoulder instability. Specifically, the exploratory research assessed whether shoulder instability patients with associated lesions such as Hill-Sachs lesions or capsular injuries had elevated biomarker levels for cartilage turnover and inflammation when compared to shoulder instability patients without the corresponding lesions. This study showed that apart from plasma COMP levels being elevated in patients with Hill-Sachs lesions, no other biomarker level was significantly different when compared across specific lesions including Hill-Sachs lesions, capsular injuries, bony Bankart lesions, and SLAP lesions. There was also no difference in biomarker levels between patients with multiple dislocations compared to patients with a first-time dislocation. Furthermore, circulating biomarker levels did not correlate with EUA instability grading at the time of surgery, suggesting that the extent or magnitude of instability also did not affect biomarker levels. The findings in this study supplement previous research showing no correlation between the number of dislocation events and the prevalence of any associated lesions [4].

Circulating protein biomarkers have been useful as a prognostic indicator of osteoarthritis progression and can be used to monitor the progress of tissue healing [17]. Paired with advanced imaging, protein biomarkers could therefore be useful in the assessment of shoulder lesions present in a shoulder instability patient. Biomarkers could provide prognostic information relative to the development of post-traumatic glenohumeral arthritis while aiding in the evaluation of the effects of therapeutic interventions in the treatment of shoulder instability, especially in evaluating whether a surgical intervention has been successful in stabilizing the shoulder and reducing the risk of long-term osteoarthritis. Although many studies have focused on the prognosis and treatment of shoulder instability and associated lesions and a handful of studies have evaluated biomarkers as predictors of developing initial shoulder instability [11, 18], it is not known whether biomarkers could help in identifying patients with shoulder instability who also have associated shoulder lesions.

COMP is a glycoprotein related to the thrombospondin family that is predominantly present in cartilage. As a marker of cartilage turnover, COMP is elevated in a variety of pathological joint conditions [8, 9, 19]. Its expression has also been identified in ligaments, tendons, menisci, and synovial membrane [20–22]. While COMP is known to help stabilize and align type II collagen molecules, the full spectrum of its functions is still unknown [23]. Following the breakdown of articular cartilage, COMP is released into the circulation, allowing the protein to be harnessed as a useful marker for collagen turnover [23]. COMP is often used as a biomarker to track the progression of osteoarthritis, and COMP elevations are associated with the formation of knee osteophytes and joint space narrowing [24, 25]. While the cellular mechanism underlying the elevation in COMP observed in Hill-Sachs lesion patients is currently unknown, the articular cartilage damage that accompanies a Hill-Sachs lesion may result in increased COMP production by cartilage and/or synovial membranes [26]. Though the effect size for COMP was medium-large, COMP likely does not hold standalone diagnostic utility above current imaging techniques or inspection under arthroscopy. Despite this, the difference in COMP levels between shoulder instability patients with and without Hill-Sachs lesions may indicate a biochemical pathological difference. To further explore this potential relationship, future research should investigate COMP markers in shoulder instability patients with and without Hill-Sachs lesions as part of large prospective cohort studies.

In addition to COMP, we evaluated the levels of inflammatory markers including HS-CRP, IL-8, and MIP-1b. HS-CRP is a marker of general systemic inflammation and is elevated in several different injuries and conditions, including in patients with anterior cruciate ligament tears, femoroacetabular impingement, and acute sciatic pain [8, 9, 27]. However, there were no differences in HS-CRP between shoulder instability patients with and without specific shoulder lesions. Interleukin 8 (IL-8) is a key mediator of inflammation in its role as a chemoattractant for neutrophils, basophils, and T cells [28, 29]. As a diagnostic marker, IL-8 has been linked with various types of inflammatory conditions [30], with IL-8 levels associated with symptom severity and pain in knee osteoarthritis [17, 31–33] as well as pain in patients with rotator cuff tears [34]. IL-8 is also elevated in shoulder capsule fibroblasts of patients with frozen shoulders [35]. However, there were no differences in IL-8 between shoulder instability patients with and without specific shoulder lesions. MIP-1b plays a major role in regulating leukocyte activation and trafficking including monocytes and T cells. Similar to IL-8, MIP-1b has been implicated in osteoarthritis with upregulation in arthritic patients [36, 37]. Circulating MIP-1b levels are also correlated with increased pain in patients with occupational shoulder and neck complaints [18]. The negative correlation between MIP-1b and the Penn shoulder suggests that MIP-1b may be involved in pathological changes associated with shoulder instability. Macrophages are known to play an essential role in both acute and chronic inflammatory conditions, including in patients with chronic rotator cuff tears [38–40]. While the source of elevated MIP-1b is not known, MIP-1b may be important in regulating and trafficking monocytes to the injured shoulder joint. A lower Penn shoulder score may suggest either early articular cartilage damage or perhaps greater shoulder instability which could be reflected in increased MIP-1b levels. Overall, the results from this study suggest that biomarkers may have limited utility as diagnostic or prognostic indicators of structural pathology in patients with shoulder instability.

The high co-occurrence of different shoulder lesions in patients with shoulder instability has been noted in previous findings [4] and in this study. Given COMP's role as a marker for cartilage turnover, the presence of multiple shoulder lesions concurrently may be expected to result in higher biomarker levels. Future large-scale studies should investigate the effect of concurrent shoulder lesions on biomarker levels. Though this study focused only on patients with traumatic anterior unidirectional shoulder instability, future studies should explore the difference in biomarker assessment between unidirectional instability and multidirectional instability (MDI). Intuitively, a higher magnitude of instability and the acuity/timing of the instability event would likely correlate with greater biomarker levels in patients with shoulder instability. However, the frequency of instability episodes may have more impact on biomarker levels than the simple diagnosis of unidirectional versus MDI. Furthermore, patients with MDI typically exhibit substantial capsular laxity [41] such that there may be minimal forces on the joint tissues with subluxation. Thus, biomarker levels may ultimately be lower in the MDI group despite such patients having presumably “more” instability compared to patients with unidirectional instability.”

There are important limitations to this exploratory study. First, we did not directly sample biomarker levels in shoulder tissue, and we acknowledge that the observed changes in systemic serum biomarkers may not be reflective of pathological changes to the unstable shoulder joint. Because serum biomarker levels would be expected to normalize over time, the absence of an elevation in serum biomarkers at the time of surgery only demonstrates that there is no sustained dysregulation detectable through serum biomarkers. Biopsies of capsular, labral, and articular cartilage tissue may provide more information about local changes in patients with instability. We attempted to perform analysis from synovial fluid, but the volume of collectable synovial fluid was too low for analysis in this patient population. Second, we chose biomarkers based on previously described roles for these proteins in cartilage turnover and inflammation, but the use of unbiased analysis strategies such as mass spectrometry (protein level) or transcriptional profiling with RNA sequencing (gene expression) may identify other biomarkers that are altered in patients with shoulder instability. Third, though time from instability event to blood draw evaluations would be expected to affect circulating biomarker levels, this data was not collected consistently across the sample of patients and was thus not included as part of the analysis. Fourth, though the results presented in this exploratory study are informative, the sample size included in this exploratory study was small, not all subjects had all markers collected and assessed, and patient comorbidities were not included in the analysis. Thus, larger trials are necessary before definitive conclusions can be made about the diagnostic and prognostic utility of protein biomarkers for patients with shoulder instability. Fifth, though different levels of patient physical activity may play a key role in biomarker measures, this study did not control for this effect or gauge the general level of physical activity beyond the standard PROM scores. In the future, large-scale studies should factor in physical activity as a control for biomarker findings. Finally, we only focused on males in this study, and while we posit that the results are applicable to women, subsequent studies that include both sexes will provide greater insight into pathological changes in patients with shoulder instability.

## 5. Conclusion

This exploratory study is the first to evaluate protein biomarker levels in the context of shoulder lesions associated with shoulder instability. The primary finding of the study is that inflammatory biomarkers including HS-CRP, IL-8, and MIP-1b were not associated with specific shoulder lesions. Biomarkers of cartilage turnover (COMP) were elevated in Hill-Sachs lesions but were not associated with any other shoulder lesions. Overall, these findings suggest that these biomarkers may have limited utility as diagnostic or prognostic indicators of shoulder lesions in patients with shoulder instability, though large-scale, longitudinal studies are still necessary to develop a more comprehensive understanding of the clinical utility of biomarkers in the assessment and management of shoulder instability.

## Figures and Tables

**Table 1:** Prevalence of associated lesions.

Type	Lesions	Total	Percentage (%)
Total patients		33	100
*Single lesion*	Bankart lesion	30	91
	Hill-sachs lesion	20	61
	Capsular injury	10	30
	Bony bankart lesion	10	30
	SLAP lesion	8	24

Co-occurrence of lesions	Bankart and hill-sachs lesion	20	61
	Bankart lesion and capsular injury	9	27
	Bankart and bony bankart lesion	7	21
	Bankart and SLAP lesion	8	24
	Hill-sachs lesion and capsular injury	6	18
	Hill-sachs and bony bankart lesion	6	18
	Hill-sachs and SLAP lesion	6	18
	Capsular injury and bony bankart lesion	3	9
	Capsular injury and SLAP lesion	6	18
	Bony bankart and SLAP lesion	2	6

## Data Availability

Due to the nature of this research, participants of this study did not agree for their data to be shared publicly, so supporting data are not publicly available, though deidentified data can be shared upon request.

## References

[1] Gottschalk L. J., Walia P., Patel R. M. (2016). Stability of the glenohumeral joint with combined humeral head and glenoid defects. *The American Journal of Sports Medicine*.

[2] Zacchilli M. A., Owens B. D. (2010). Epidemiology of shoulder dislocations presenting to emergency departments in the United States. *Journal of Bone and Joint Surgery American Volume*.

[3] Calandra J. J., Baker C. L., Uribe J. (1989). The incidence of Hill-Sachs lesions in initial anterior shoulder dislocations. *Arthroscopy: The Journal of Arthroscopic & Related Surgery*.

[4] Carrazzone O. L., Tamaoki M. J. S., Ambra L. F. M., Neto N. A., Matsumoto M. H., Belloti J. C. (2011). Prevalence OF lesions associated with traumatic recurrent shoulder dislocation. *Revista Brasileira de Ortopedia (English Edition)*.

[5] Provencher M. T., Frank R. M., LeClere L. E. (2012). The Hill-Sachs lesion: diagnosis, classification, and management. *Journal of the American Academy of Orthopaedic Surgeons*.

[6] Rowe C. R., Zarins B., Ciullo J. V. (1984). Recurrent anterior dislocation of the shoulder after surgical repair. Apparent causes of failure and treatment. *The Journal of Bone and Joint Surgery*.

[7] Burkhart S. S., De Beer J. F. (2000). Traumatic glenohumeral bone defects and their relationship to failure of arthroscopic Bankart repairs. *Arthroscopy: The Journal of Arthroscopic & Related Surgery*.

[8] Bedi A., Lynch E. B., Sibilsky Enselman E. R. (2013). Elevation in circulating biomarkers of cartilage damage and inflammation in athletes with femoroacetabular impingement. *The American Journal of Sports Medicine*.

[9] Mendias C. L., Lynch E. B., Davis M. E. (2013). Changes in circulating biomarkers of muscle atrophy, inflammation, and cartilage turnover in patients undergoing anterior cruciate ligament reconstruction and rehabilitation. *The American Journal of Sports Medicine*.

[10] Neuman P., Dahlberg L. E., Englund M., Struglics A. (2017). Concentrations of synovial fluid biomarkers and the prediction of knee osteoarthritis 16 years after anterior cruciate ligament injury. *Osteoarthritis and Cartilage*.

[11] Owens B. D., Cameron K. L., Bokshan S. L., Clifton K. B., Svoboda S. J., Wolf J. M. (2017). Serum cartilage biomarkers and shoulder instability. *Orthopedics*.

[12] Leggin B. G., Michener L. A., Shaffer M. A., Brenneman S. K., Iannotti J. P., Williams G. R. (2006). The Penn shoulder score: reliability and validity. *Journal of Orthopaedic & Sports Physical Therapy*.

[13] Richards R. R., An K.-N., Bigliani L. U. (1994). A standardized method for the assessment of shoulder function. *Journal of Shoulder and Elbow Surgery*.

[14] Mendias C. L., Lynch E. B., Gumucio J. P. (2015). Changes in skeletal muscle and tendon structure and function following genetic inactivation of myostatin in rats. *The Journal of Physiology*.

[15] Mendias C. L., Schwartz A. J., Grekin J. A., Gumucio J. P., Sugg K. B. (2017). Changes in muscle fiber contractility and extracellular matrix production during skeletal muscle hypertrophy. *Journal of Applied Physiology*.

[16] McGrath R. E., Meyer G. J. (2006). When effect sizes disagree: the case of r and d. *Psychological Methods*.

[17] Haraden C. A., Huebner J. L., Hsueh M.-F., Li Y.-J., Kraus V. B. (2019). Synovial fluid biomarkers associated with osteoarthritis severity reflect macrophage and neutrophil related inflammation. *Arthritis Research and Therapy*.

[18] Matute Wilander A., Kåredal M., Axmon A., Nordander C. (2014). Inflammatory biomarkers in serum in subjects with and without work related neck/shoulder complaints. *BMC Musculoskeletal Disorders*.

[19] Daghestani H. N., Kraus V. B. (2015). Inflammatory biomarkers in osteoarthritis. *Osteoarthritis and Cartilage*.

[20] Clark A. G., Jordan J. M., Vilim V. (1999). Serum cartilage oligomeric matrix protein reflects osteoarthritis presence and severity: the Johnston County Osteoarthritis Project. *Arthritis & Rheumatism*.

[21] Müller G., Michel A., Altenburg E. (1998). COMP (cartilage oligomeric matrix protein) is synthesized in ligament, tendon, meniscus, and articular cartilage. *Connective Tissue Research*.

[22] Neidhart M., Hauser N., Paulsson M., DiCesare P. E., Michel B. A., Hauselmann H. J. (1997). Small fragments of cartilage oligomeric matrix protein in synovial fluid and serum as markers for cartilage degradation. *Rheumatology*.

[23] Garvican E. R., Vaughan-Thomas A., Clegg P. D., Innes J. F. (2010). Biomarkers of cartilage turnover. Part 2: non-collagenous markers. *The Veterinary Journal*.

[24] Bi X. (2018). Correlation of serum cartilage oligomeric matrix protein with knee osteoarthritis diagnosis: a meta-analysis. *Journal of Orthopaedic Surgery and Research*.

[25] Golightly Y. M., Marshall S. W., Kraus V. B. (2011). Biomarkers of incident radiographic knee osteoarthritis: do they vary by chronic knee symptoms?. *Arthritis & Rheumatism*.

[26] Orvets N. D., Parisien R. L., Curry E. J. (2017). Acute versus delayed magnetic resonance imaging and associated abnormalities in traumatic anterior shoulder dislocations. *Orthopaedic Journal of Sports Medicine*.

[27] Sturmer T., Raum E., Buchner M. (2005). Pain and high sensitivity C reactive protein in patients with chronic low back pain and acute sciatic pain. *Annals of the Rheumatic Diseases*.

[28] Bickel M. (1993). The role of interleukin-8 in inflammation and mechanisms of regulation. *Journal of Periodontology*.

[29] Zhang W., Chen H. (2002). [The study on the interleukin-8 (IL-8)]. *Sheng Wu Yi Xue Gong Cheng Xue Za Zhi*.

[30] Shahzad A., Knapp M., Lang I., Köhler G. (2010). Interleukin 8 (IL-8)-a universal biomarker?. *International Archives of Medicine*.

[31] Kaneko S., Satoh T., Chiba J., Ju C., Inoue K., Kagawa J. (2000). Interleukin-6 and interleukin-8 levels in serum and synovial fluid of patients with osteoarthritis. *Cytokines, Cellular & Molecular Therapy*.

[32] Monibi F., Roller B., Stoker A., Garner B., Bal S., Cook J. (2016). Identification of synovial fluid biomarkers for knee osteoarthritis and correlation with radiographic assessment. *Journal of Knee Surgery*.

[33] Ruan G., Xu J., Wang K. (2019). Associations between serum IL-8 and knee symptoms, joint structures, and cartilage or bone biomarkers in patients with knee osteoarthritis. *Clinical Rheumatology*.

[34] Okamura K., Kobayashi T., Yamamoto A. (2017). Shoulder pain and intra-articular interleukin-8 levels in patients with rotator cuff tears. *International Journal of Rheumatic Diseases*.

[35] Akbar M., McLean M., Garcia-Melchor E. (2019). Fibroblast activation and inflammation in frozen shoulder. *PLoS One*.

[36] Menten P., Wuyts A., Van Damme J. (2002). Macrophage inflammatory protein-1. *Cytokine & Growth Factor Reviews*.

[37] Patel D. D., Zachariah J. P., Whichard L. P. (2001). CXCR3 and CCR5 ligands in rheumatoid arthritis synovium. *Clinical Immunology*.

[38] Dakin S. G., Martinez F. O., Yapp C. (2015). Inflammation activation and resolution in human tendon disease. *Science Translational Medicine*.

[39] Mendias C. L., Roche S. M., Harning J. A. (2015). Reduced muscle fiber force production and disrupted myofibril architecture in patients with chronic rotator cuff tears. *Journal of Shoulder and Elbow Surgery*.

[40] Thomopoulos S., Parks W. C., Rifkin D. B., Derwin K. A. (2015). Mechanisms of tendon injury and repair. *Journal of Orthopaedic Research*.

[41] Warby S. A., Watson L., Ford J. J., Hahne A. J., Pizzari T. (2017). Multidirectional instability of the glenohumeral joint: etiology, classification, assessment, and management. *Journal of Hand Therapy*.

